# Overexpression of *Physcomitrium patens* cell cycle regulators leads to larger gametophytes

**DOI:** 10.1038/s41598-023-31417-x

**Published:** 2023-03-15

**Authors:** Anantha Peramuna, Carmen Quiñonero López, Francisco Javier Astorga Rios, Hansol Bae, Jonatan Ulrik Fangel, Rituraj Batth, Jesper Harholt, Henrik Toft Simonsen

**Affiliations:** 1 Department of Biotechnology and Biomedicine, Technical University of Denmark, Kongens Lyngby, Denmark; 2 Plant and Environmental Science, University of Copenhagen, Copenhagen, Denmark; 3 Carlsberg Research Laboratories, Copenhagen, Denmark; 4 Laboratoire Biotechnologies Végétales Plantes Aromatiques et Médicinales, Faculté des Sciences, Université Jean Monnet, 23 Rue Dr Paul Michelon, 42023 Saint-Etienne Cédex 2, France

**Keywords:** Plant biotechnology, Plant biotechnology, Plant physiology

## Abstract

Regulation of cell division is crucial for the development of multicellular organisms, and in plants, this is in part regulated by the D-type cyclins (CYCD) and cyclin-dependent kinase A (CDKA) complex. Cell division regulation in *Physcomitrium* differs from other plants, by having cell division checks at both the G1 to S and G2 to M transition, controlled by the CYCD1/CDKA2 and CYCD2/CDKA1 complexes, respectively. This led us to hypothesize that upregulation of cell division could be archived in Bryophytes, without the devastating phenotypes observed in Arabidopsis. Overexpressing lines of *PpCYCD1*, *PpCYCD2*, *PpCDKA1*, or *PpCDKA2* under *Ubiquitin* promotor control provided transcriptomic and phenotypical data that confirmed their involvement in the G1 to S or G2 to M transition control. Interestingly, combinatorial overexpression of all four genes produced plants with dominant PpCDKA2 and PpCYCD1 phenotypes and led to plants with twice as large gametophores. No detrimental phenotypes were observed in this line and two of the major carbon sinks in plants, the cell wall and starch, were unaffected by the increased growth rate. These results show that the cell cycle characteristics of *P. patens* can be manipulated by the ectopic expression of cell cycle regulators.

## Introduction

Bryophytes, including the moss *Physcomitrium patens* (syn. *Physcomitrella patens*), spend the majority of their life as photoautotrophic haploid gametophytes. Under appropriate environmental conditions, diploid sporophytes develop from leafy gametophores and produce spores and continue the dominant haploid life cycle. Haploid gametophyte cells in *P. patens* exist in two physiologically distinct cell types: the filamentous juvenile form of protonema, consisting of apically growing chloronema and caulonema cells and the adult leafy structure of gametophores. Protonema cells differentiate into adult leafy gametophores by initiating the division of three-faced apical cells (bud)^1–3^.

A dividing cell goes through four stages: G1, S, G2, and M phase, followed by cytoplasm division to separate the two daughter cells. The DNA replicates in S phase, with subsequent segregation in the M phase. The progression from G1 to S and G2 to M phases is controlled by cyclins and cyclin-dependent kinases (CDK) to ensure the completion of the previous S and M phases^4^. Plant cells are under normal conditions arrested at the G1-S transition and re-enter cell cycle progression upon proper stimuli^4,5^. In *P. patens*, D-type cyclins and CDKA forms complexes upon external or internal stimuli by sucrose, auxins, or cytokinin’s to progress the cell cycle through the G1 phase. Activation of the CYC/CDK complex phosphorylates the negative cell cycle regulator known as retinoblastoma (Rb) proteins to relieve the repression of E2F transcription factors, which is required for the S phase entry^4^. To follow the progression of the cell division an array of genes has been identified as G1-S and G2-M phase markers. Among these are the early S phase marker *HFO* (histone H4 gene), which is expressed just at the start of the S phase and marks the progression through the G1 to S. For the G2-M two classic markers are *CYCB1* + *2* that both are expressed during the G2 to M phase. All of these have been used to study the cell division in plants and are good markers of the phase changes^6^. All three genes are used in this study.

*P. patens* protonema cells display a distinctive tissue-specific cell cycle alteration as compared to other plants. The faster-growing caulonema cells are arrested at G1-S transition controlled by CYCD1/CDKA2, whereas the slower-growing chloronema cells^7^ are arrested at G2-M transition controlled by CYCD2/CDKA1^3,5^. This unique cell cycle arrest facilitates the highly efficient homologous recombination which is unique for bryophytes and not seen among other land plants^8–11^.

Fast cell proliferation is normally beneficial in industrial biotechnology, and *P. patens* is today used for biopharmaceutical production^10,12–14^. Although a lot of work has been done on ectopic expression of CYCD and CDKA proteins in vascular plants^15–17^, these efforts have led to detrimental phenotypes. Here we enhanced cell proliferation through ectopic expression of endogenous *PpCDKA1*, *PpCDKA2*, *PpCYCD1* and *PpCYCD2* genes individually and all four together. This led to an increase in gametophore size, which offer new opportunities for industrial biotechnology. Additionally, this showed that the transition from G1 to S is controlled by CYCD1/CDKA2, and the transition from G2 to M is controlled by CYCD2/CDKA1.

## Results and discussion

### Effects of PpCDKA1 overexpression

**Figure 1:**
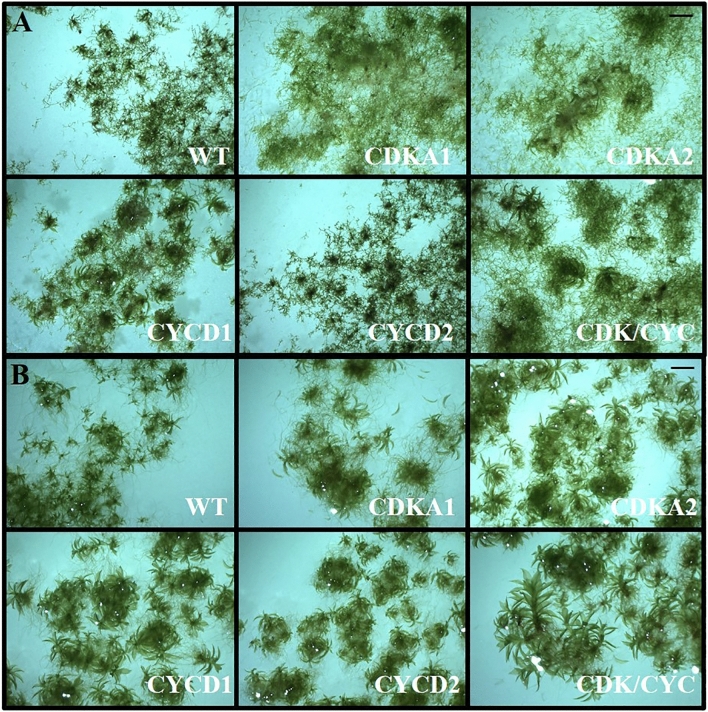
Phenotypic analysis of WT and transgenic lines. (**A**) Cell lines were grown in BCDAT medium for 35 days and photographed using Miotic SMZ-171 microscope. (**B**) Cells grown in similar conditions as above in BCD media. The cell lines are *ZmUbi:PpCDKA1* (CDKA1)*, ZmUbi:PpCDKA2* (CDKA2)*, ZmUbi:PpCYCD1* (CYCD1)*, ZmUbi:PpCYCD2* (CYCD2)*, ZmUbi:PpCDKA1-PpCDKA2-OsAct:PpCYCD2-PpCYCD1* (CDK/CYC) and wildtype (WT). Same scale bar for all pictures = 1.5 mm.

*PpCDKA1* overexpression promoted protonema cell growth in BCDAT medium though with fewer as well as 10% shorter gametophores (Fig. 1A). The cells of the gametophores were 15% smaller, possibly explaining the observed size difference (Table 1A). When *ZmUbi:PpCDKA1* lines were grown on solid BCD media it led to some differentiation of protonema into gametophores. The gametophores were 6% shorter and had 12% larger cell size, thus shorter with bigger and fewer cells. This indicates that *ZmUbi:PpCDKA1* influences cell division negatively (Fig. 1B, Table 1B).

**Table 1:** Comparison of cell area and gametophore height of WT and transgenic cell lines.

Cell line	Cell area × 10^–3^ (mm^2^)	% change	Gametophore height (mm)	% change
(A) Grown in BCDAT medium				
WT	1.10 ± 3.35E−02		0.40 ± 0.01	
CDKA1	0.94 ± 2.74E−02	−15.31*	0.36 ± 0.01	−10
CDKA2	1.02 ± 4.65E−02	−7.91	0.64 ± 0.01	60*
CYCD1	1.02 ± 3.09E−02	−7.64	0.65 ± 0.02	62.5*
CYCD2	0.68 ± 2.83E−02	−38.23*	0.287 ± 0.01	−28.25*
CDK/CYC	1.18 ± 3.75E−02	6.48	0.78 ± 0.03	95*
(B) Grown in BCD medium				
WT	0.92 ± 3.15E−02		0.48 ± 0.01	
CDKA1	1.04 ± 4.23E−02	12.28	0.45 ± 0.02	−6.25
CDKA2	1.06 ± 3.94E−02	15.24	0.76 ± 0.02	58.33*
CYCD1	1.24 ± 4.42E−02	34.68	0.81 ± 0.02	68.75*
CYCD2	0.83 ± 1.96E−02	-9.63	0.47 ± 0.01	−2.08
CDK/CYC	1.09 ± 5.36E−02	18.19	0.95 ± 0.02	97.92*

The cell lines are *ZmUbi:PpCDKA1* (CDKA1)*, ZmUbi:PpCDKA2* (CDKA2)*, ZmUbi:PpCYCD1* (CYCD1)*, ZmUbi:PpCYCD2* (CYCD2)*, ZmUbi:PpCDKA1-PpCDKA2-OsAct:PpCYCD2-PpCYCD1* (CDK/CYC) and wildtype (WT). Cell area and gametophore height of cells (n > 50) grown in (A) BCDAT medium, (B) BCD medium for 35 days. % change represents the cell area and gametophore height difference of WT to the transgenic lines. Significance of the data was determined by one-way ANOVA test; P < 0.001 marked with an *.

**Figure 2:**
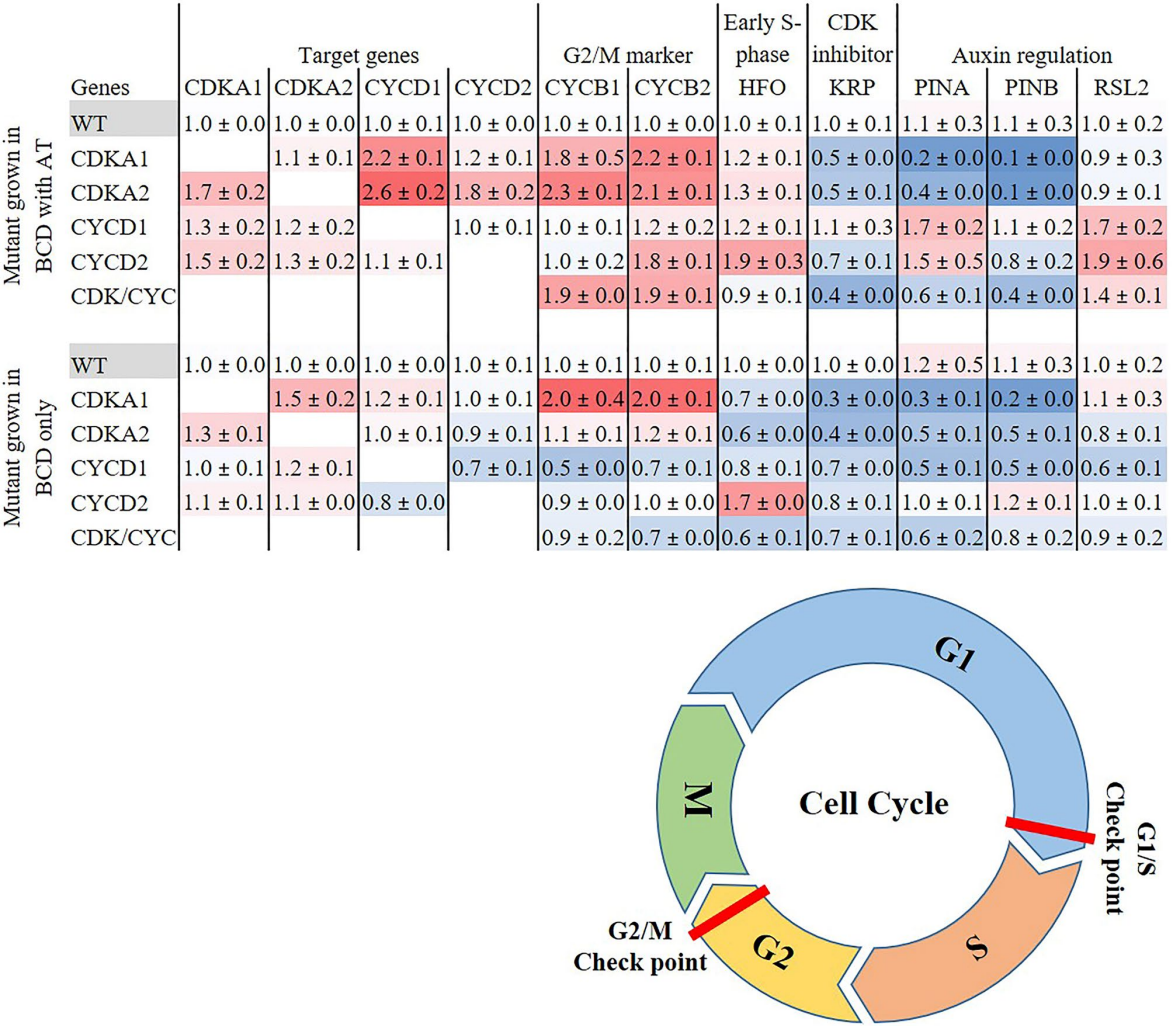
Heat Map of relative expression of cell cycle and cell development markers in mutant lines normalized to actin and tubulin. All cell lines, *ZmUbi:PpCDKA1* (CDKA1)*, ZmUbi:PpCDKA2* (CDKA2)*, ZmUbi:PpCYCD1* (CYCD1)*, ZmUbi:PpCYCD2* (CYCD2)*, ZmUbi:PpCDKA1-PpCDKA2-OsAct:PpCYCD2-PpCYCD1* (CDK/CYC), are grown in BCDAT and BCD media, respectively. The figure also illustrates the cell cycle phases with the important checkpoints. The genes tested are CDKA1, CDKA2, Cyclin B1 and B2 (CYCB1, CYCB2), CYCD1, CYCD2, histone H4 gene (HFO), KIP-RELATED PROTEIN (KRP), auxin efflux carriers PINA and PINB, and auxin-induced transcription factor RSL2. The figure also illustrates the cell cycle phases with the important checkpoints. The G1/S marker is the HFO gene, and G2/M marker is the CYCB1 + 2.

An isolated *ZmUbi:PpCDKA1* gametophyte grown for 35 days in liquid BCD medium was dominant in protonema cells and without larger gametophores, unlike WT that developed abundant amounts of gametophores (Fig. 1, 2, and Supplementary Fig. S1). The lesser gametophore differentiation in the *ZmUbi:PpCDKA1* line prompted us to look at the expression of *PpPIN*, involved in auxin efflux^18,19^, as well as auxin gradient formation in the apical tip. PIN is essential for the initiation of protonemal differentiation, and it is established that auxin transport by PIN regulates gametophyte differentiations in moss^2,19,20^. qPCR analysis of *PpPINA* and *PpPINB* transcripts in *ZmUbi:PpCDKA1* showed a > 80% reduction in both BCD and BCDAT media (Fig. 2). The lack of protonema differentiation towards gametophores as seen in Figs. 1, 2 and supplementary figures and stunted and lesser gametophore development in *ZmUbi:PpCDKA1* lines can among other things be attributed to down-regulation of *PIN* and thereby change in auxin levels. However, the relationship between *CDKA1* overexpression and *PIN* down-regulation is still unknown. No apparent change in the expression of auxin-induced transcription factors like *RSL2*^21^ was observed in *ZmUbi:PpCDKA1* in either of the two media (Fig. 2).

Transcript analysis in BCDAT media showed that the transcript levels of the G2 to M phase markers, *CYCB1* and *CYCB2*^4,5^ were increased by 80% and 121%. Thus, *CDKA1* is likely to be involved in the G2 to M transition as previously suggested^5,7^.

### Effects of PpCDKA2 overexpression

**Figure 3:**
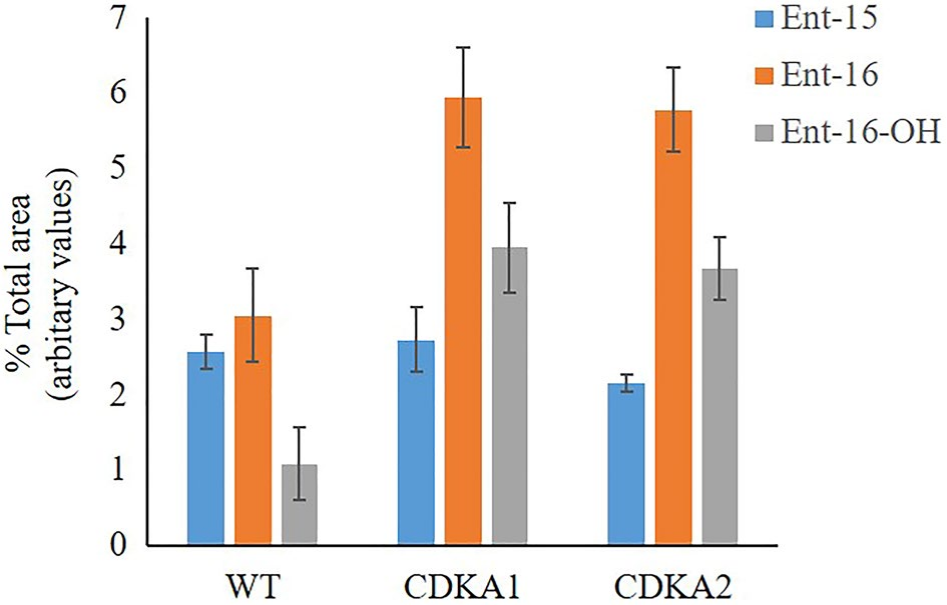
GC/MS analysis of cell *ent*-kaurene and *ent*-kaurenoic acid levels. The graph represents the area of *ent*-kaur-15ene, *ent*-kaur-16-ene and 16-hydroxy-*ent*-kaurene peaks to the total peaks of the chromatogram (n = 3).

Like *ZmUbi:PpCDKA1*, single *ZmUbi:PpCDKA2* gametophores grown on BCD media were dominant in protonema cells and with small gametophores (Fig. 1, and Supplementary Fig. S1),. Furthermore, *PpPIN* expression was suppressed, but not as severely as in *ZmUbi:PpCDKA1*, possibly explaining the development of small gametophores as was not seen with *ZmUbi:PpCDKA1* (Figs. 1 and 3). This indicates a different mechanism of the two cyclin-dependent kinases (Table 1).

The transcript analysis of *ZmUbi:PpCDKA2*, showed effects on cell cycle progression through G1 phase by increased expression of *PpCDKA1*, *PpCYCD1*, *PpCYCD2* and down-regulation of *PpKRP* (Fig. 2). Signs of cell cycle advancement through G2-M phase are observed with increased expression of *PpCYCB1* and *PpCYCB2*. Considering the protonema-dominant phenotype of the *ZmUbi:PpCDKA2* lines, transcript analysis of cells grown in BCDAT suggests that ectopic expression of *PpCDKA2* leads to protonema-specific gene expression and cell cycle proliferation. The up-regulation of G1-S and G2-M markers were more pronounced when cells were grown in BCDAT than in BCD media (Fig. 2). This shows that the ectopic expression of *PpCDKA2* counteracts the ammonium tartrate G1-S inhibition, and links the *PpCDKA2* to the G1 to S transition.

It has been demonstrated that *ent*-kaurenoic acid derived from *ent*-kaurene positively regulates the chloronema to caulonema differentiation as well as spore development in *P. patens*^22,23^. The two protonemal dominant lines *ZmUbi:PpCDKA1* and *ZmUbi:PpCDKA2* were grown on BCDAT for 28 days and compared to WT by a GC–MS analysis for the *ent-*kaurene level in the whole of the moss culture, thus both protonemal and the small possible gametophore tissue. The analysis showed for both *ZmUbi:PpCDKA1* and *ZmUbi:PpCDKA2* a significant increase in the levels of *ent*-kaurenes and *ent*-kaurenoic acid (Fig. 3). This indicates that the plants would try to compensate for the suppressed cell differentiation with the expression of other differentiation signals and hormones such as the *ent-*kaurene, highlighting the plasticity of plant cells to utilize several different signal pathways to obtain the same phenotype.

### Effects of CYCD1 overexpression

Ectopic expression of *PpCYCD1* promoted gametophore development and less protonema tissue in both BCD and BCDAT (Figs. 1, 2). The *ZmUbi:PpCYCD1* gametophores grown in BCD and BCDAT were 1.7 and 1.6 times taller; and with larger leaf cell area on BCD media when compared to the WT (Table 1). The significant growth in gametophore height can in part be related to the increased expression of *PpPINA* and *PpRSL2* transcript levels (Fig. 2), which regulates the differentiations into gametophores and more new buds as also seen in *Arabidopsis*^17^.

The G2-M markers *CYCB1* + *2,* the CDKA/CYCD complex suppresser *KRP* (KIP-RELATED PROTEIN) and early S phase marker *HFO* (histone H4 gene) were significantly downregulated in *ZmUbi:PpCYCD1* cells grown on BCD (Fig. 2), whereas no significant differences were observed in cells on BCDAT (Fig. 2). A previous finding of *P. patens* gametophores hypothesized that gametophore cells tend to arrest in the late S phase^5^, and the observed increase in the size and numbers of gametophore cells together with low expression of *CYCB1* + *2, HFO* supports the previous finding. Overall these observations show that *PpCYCD1* is likely to be involved in the G1 to S phase transition.

The gametophore cell area increased for all *ZmUbi:PpCDKAs* and *ZmUbi:PpCYCD1* lines grown in BCD media. The *ZmUbi:PpCYCD1* line had the most significant increase in gametophore cell area of 35% (Table 1). The cell area growth in plants could be under a cell cycle-related or cell cycle-independent regulation^16^. The increase in cell size suggests that CYCD1 might be able to influence the gametophore cell area growth in a cell cycle-related manner, but again this requires further studies.

### Effects of CYCD2 overexpression

Similar to *ZmUbi:PpCYCD1*, the induction of gametophores in the *ZmUbi:CYCD2* line was not impaired (Fig. 1). However, the height and cell area were reduced as compared to all the other lines in this study (Table 1). On average *ZmUbi:PpCYCD2* grown on BCADT had 38% smaller gametophore cell size and 28% shorter plants (Table 1). When grown on BCD the cell size was reduced by 10%. No significant differences were observed in *PIN* transcript levels on any of the media, whereas *RSL2* was upregulated on BCD but not BCDAT (Fig. 2). This indicates a link between auxin differentiation signals like RSL2^21^ and cell cycle progression with the inhibition by ammonium tartrate. The data also suggest that Cyclin D’s influence the gametophore cell size in a cell-cycle dependent manner as previously speculated^16^.

The early S phase marker (Histone H4 gene, HFO) was upregulated on both BCDAT and BCD by 92% and 70% in *ZmUbi:PpCYCD2* line (Fig. 2). This shows a relative increase in early S phase cells and that the gametophores were not arrested in the late S phase as seen in WT and *ZmUbi:PpCYCD1*, suggesting that *PpCYCD2* induce the G2 to M transition.

Over-expression of *AtCYCD3;1* in *Arabidopsis* has similar phenotypes as induced by exogenous cytokinin^24^. Previously, it has been shown that the daily replacement of the media stunted the differentiation of *P. patens* cells due to the removal of secreted auxins, cytokinins and other hormones^3^. To observe if overexpression of either *PpCYCD1* or *PpCYCD2* have any such phenotypic effect, we replaced the growth medium every day for two weeks. WT, *ZmUbi:PpCYCD1* and *ZmUbi:PpCYCD2* lines were all equally delayed when media was replaced every day (data not shown) indicating that *PpCYCD1* and *PpCYCD2* overexpression cannot replace the effect of secreted hormones.

### Overexpression of CDKA1, CDKA2, CYCD1 and CYCD2 in the same cell line (CDK/CYC)

The unique functions of CDKAs and CYCDs prompted us to introduce CDKA1, CDKA2, CYCD1 and CYCD2 in the same transformation event to study the additive effects in *P. patens* and the potential for industrial application. The *ZmUbi:PpCDKA1-PpCDKA2-OsAct:PpCYCD2-PpCYCD1* line showed a pronounced effect in both gametophore height (twofold) and gametophore cell area independent of growth media (Tables 1 and 2).

**Table 2:** Comparison of cell area and gametophore height of WT and transgenic cell lines.

Cell line	Rhizoid length (mm)	“Leaf” length (mm)	“Leaf” width (mm)	Gametophore height (mm)	Dry weight increase (mg)
WT	0.76 ± 0.217	0.07 ± 0.0011	0.015 ± 0.003	0.55 ± 0.007	2328*
CDK/CYC	0.57 ± 0.143*	0.08 ± 0.0011	0.026 ± 0.002*	1.05 ± 0.016* (+ 90%)	1080

Rhizoid, “leaf” and gametophore height of cells (n > 10) grown in BCD medium for 90 days. The dry weight started at 410 mg (WT) and 440 mg (CDK/CYC). Significance of the data was determined by one-way ANOVA test; P < 0.001 marked with an *.

Analysis of cell cycle-related transcripts showed over 86% increase in both *PpCYCB1* + *2* and 63% relatively low expression of *PpKRP* when grown on BCDAT, but not in BCD for *PpCYCB1* + *2* though still with a 30% reduction *PpKRP*. For both media, the early S phase *PpHFO* was reduced, indicating fewer cells in the S phase. When all four genes are overexpressed in CDK/CYC the results suggest that transgenic cells advance faster through all the stages of the cell cycle (Fig. 2, Supplementary Fig. S2).

**Figure 4:**
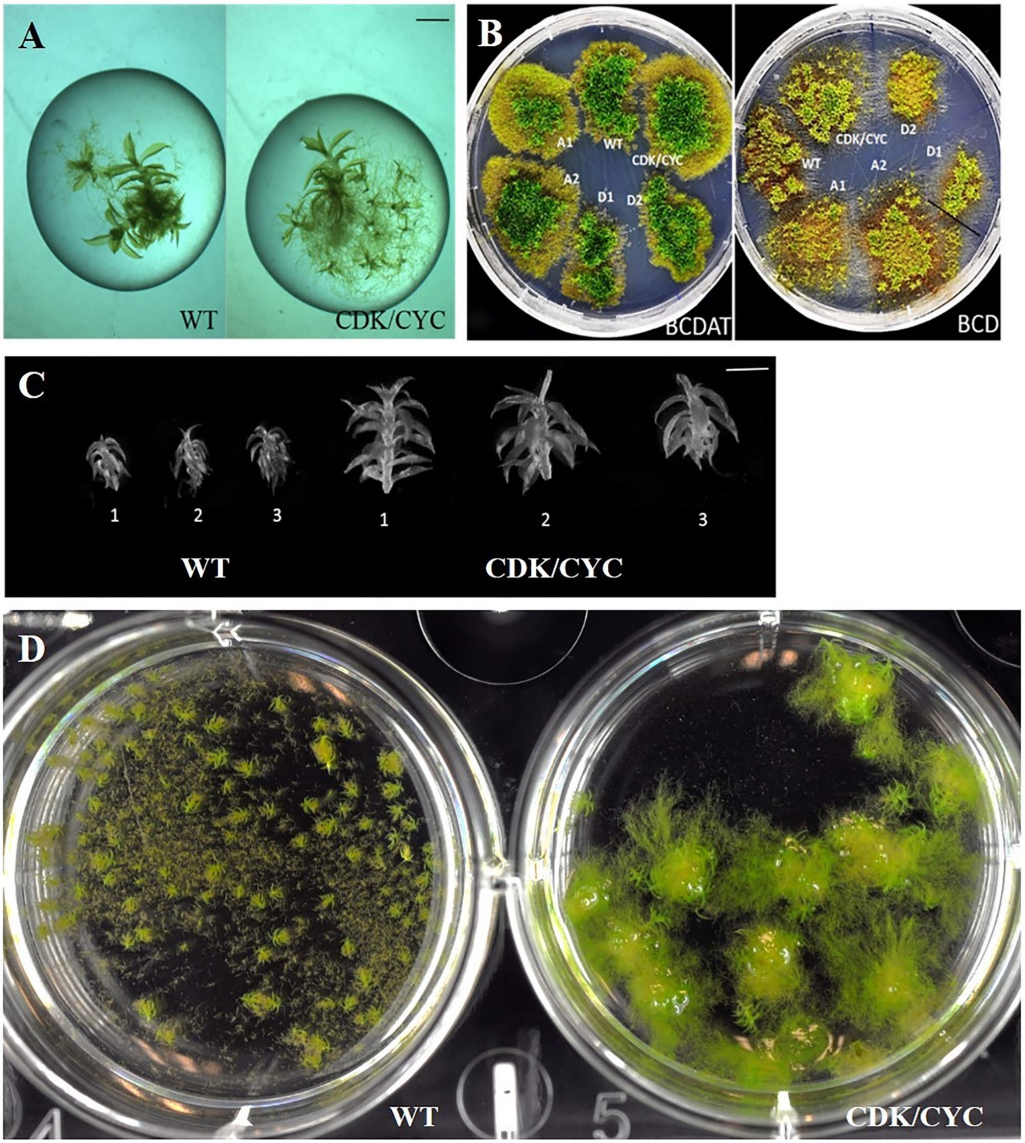
(**A**) Comparison of a single gametophore of WT and the transgenic line grown in BCD liquid medium for 35 days (scale bar = 0.6 mm). (**B**) Homogenized cell lines studied in this work were grown for 90 days on BCDAT and BCD solid media for phenotypic comparison. (**C**) Comparison of gametophore phenotype of WT and *ZmUbi:PpCKA1–PpCDKA2–OsAct:PpCYCD2–PpCYCD1* biological triplicate lines grown under same conditions for 35 days (scale bar = 0.6 mm). (**D**) Comparison of a single gametophore of WT and *ZmUbi:PpCKA1–PpCDKA2–OsAct:PpCYCD2–PpCYCD1* grown in BCD liquid medium for 90 days, in six well microtiter plates.

The dominating effects of CDKA2 and CYCD1 masked the dwarf gametophore phenotype of CDKA1 and CYCD2 when the CDK/CYC transgenic line was grown on both BCD and BCDAT mediums (Fig. 4A,B). Due to the large gametophore size of this transgenic line (Fig. 4C), the 90 days old “leaves” size were measured and the “leaves” were up to 70% broader (Table 2). Growing the CDK/CYC transgenic line for 90 days line in liquid BCD media yielded about half the biomass of WT when grown in BCD (Table 2). The major source for the difference between the two lines is the number and size of the rhizoids. Unlike with the gametophytes we observed only very few rhizoids in the CDK/CYC transgenic moss, and the length (Table 2, Fig. 4D) and numbers of rhizoids are significantly lower for the CDK/CYC transgenic line when grown in liquid culture (Figs. 1B, 4D and Supplementary Figs. S1 and S2).

This large phenotype of gametophores of *ZmUbi:PpCDKA1-PpCDKA2-OsAct:PpCYCD2-PpCYCD1* (CDK/CYC) prompted us to analyse the cell wall composition and the starch content of all the transgenic lines reported in this study. No significant differences in the cell wall composition were observed between WT and the transgenic lines. The subsequent analysis of starch content showed that none of the engineered strains had reduced starch content when compared to WT. The *ZmUbi:PpCDKA1* line had a surprising 50% increase in starch content over WT (Supplementary Figs. S3, S4 and Table S1). Thus, none of the transgenic lines appeared energy starved despite the growth phenotypes.

Ubiquitous expression of CYCD1, CYCD2, CDKA1 and CDKA2 led to more pleiotropic effects, for example, differences in cell differentiation leading to fewer gametophores for CDKA1, as discussed above. Based on these findings we tested combined overexpression of the four *P. patens* genes *CDKA1*, *CDKA2*, *CYCD1*, and *CYCD2* and could observe that it leads to a twofold increase in the height of the gametophores but significantly smaller rhizoids and thus less accumulation of biomass. It remains to be investigated how the increase in gametophore size can be adopted in the rhizoids and this way provide significantly more biomass. The study confirms the link between the cyclins and cyclin-dependent kinases and the cell cycle phases. This was previously investigated through knock-outs, but our study confirms this through overexpression of the genes. The study shows that upregulation of the cell cycle does not always lead to dwarf mutants when the upregulation is performed through several genes, and not only one target. Similar studies remain to be done in other plants due to the technical challenges with the overexpression of multiple genes in vascular plants. Overall the study opens up for more questions on how the regulation of the cell cycle can be manipulated and show that with several genes uncoupled from the native regulation the cell cycle can be positively manipulated.

## Conclusion

Combined overexpression was tested of the four *P. patens* genes *CDKA1*, *CDKA2*, *CYCD1*, and *CYCD2* showed that it leads to a twofold increase in the height of the plants and increased accumulation of biomass in the early phase, but over a short time to less biomass, as the rhizoids growth was stunted. Individual overexpressing lines of *PpCYCD1*, *PpCYCD2*, *PpCDKA1*, or *PpCDKA2* provided transcriptomic and phenotypical data that confirmed their involvement cell division cycle, and that the CYCD1/CDKA2 and CYCD2/CDKA1 complexes regulate the G1 to S and G2 to M phase transition, respectively.

## Material and methods

### Plant material, growth conditions, and transformation

Wild-type *Physcomitrium patens* (syn. *Physcomitrella patens*)^25^ (Gransden ecotype) was obtained from the International Moss Stock Center at the University of Freiburg (http://www.moss-stock-center.org/). All material used comply with relevant institutional, national, and international guidelines and legislation. Cells were grown in continuous white and red light at 23 °C, in BCD liquid or solid media with nitrate as the nitrogen source or ammonium tartrate as the additional nitrogen source (BCDAT). Further information on growth conditions and transformation processes could be found in previous publications^11,26,27^.

### Strain construction

*P. patens* cells were transformed according to previously published protocols with minor modifications^11,26^ and yielded following moss cell mutant lines*. ZmUbi:PpCDKA1, ZmUbi:PpCDKA2, ZmUbi:PpCycD1, ZmUbi:PpCycD2, ZmUbi:PpCDKA1-PpCDKA2-OsAct:PpCYCD2-PpCYCD1*.

### DNA fragments and genes

The coding sequence (CDS^5^) of *PpCDKA1* (AJ515321), *PpCDKA2* (AB547329), *PpCYCD1* (AJ428953) and *PpCYCD2* (AB547332) was PCR amplified from the cDNA of *P. patens* and stably transformed into the Pp108 neutral locus individually with the ubiquitin promoter and the OCS terminator. Further, CDS of *PpCDKA1* and *PpCDKA2*, combined with translationally cleaved LP4/2A linker^28,29^ was synthesized as one construct using Thermo Fisher Scientific gene synthesis services. Similarly, *PpCYCD1* and *PpCYCD2* combined with LP4/2A linker were synthesized as *PpCYCD2–PpCYCD1*. Subsequently, all four genes were transformed into the Pp108 neutral locus using the ubiquitin and actin promoters. The *Zea mays* ubiquitin promoter, rice actin promoter, and the G418 antibiotic cassette was amplified from the pMP1355 and pZAG1 vector^28,29^. Stable transformants for each construct were selected according to previously published methods^11,26,28^. The list of the primers used is listed in Supplementary Table S2, S3, S4 and S5.

### Phenotypic analysis

*ZmUbi:PpCDKA1, ZmUbi:PpCDKA2, ZmUbi:PpCYCD1, ZmUbi:PpCYCD2, ZmUbi:PpCDKA1, ZmUbi:PpCDKA1-PpCDKA2-OsAct:PpCYCD2-PpCYCD1* and WT cell lines were blended and continuously grown in triplicates in BCD and BCDAT medium for 35 days. The media was changed at 17 days. The phenotype of each cell line was assessed by comparing images obtained with a Moticam 5.0 MP camera mounted on a Miotic SMZ-171 microscope. A selection of the pictures are found in Figs. 1 and 2, and Supplementary Figs. S1 and S2.

### Cell size, gametophore height and “leaf” perimeter measurements

Cell size and gametophore height were measured by imaging leafy gametophore tissues of cells grown in continuous BCD and BCDAT liquid medium, the measurements were based on triplicates. For cell size measurements two trials were conducted with two different timelines, one at 35 days for all mutants (Table 1) and one at 90 days for *ZmUbi:PpCDKA1–PpCDKA2–OsAct:PpCYCD2–PpCYCD1* (Table 2). Wild type was included in both experiments.

The mid-section of the larger “leaves” of the gametophores was imaged under the 200× magnification with Nikon ECLIPSE E1000 microscope. The tissue is not actual leaves but looks like it and as such referred to as “leaf” or leafy structures. Further, the micrographs were processed with the ImageJ software to calculate the area of the gametophore cells. From each gametophore, at least eight cells were measured. Gametophore height, rhizoid length and “leaf” length and width were measured by imaging with a Miotic SMZ-171 microscope equipped with a Moticam 5.0 MP camera. The single gametophytes were placed in a drop of media for easier analysis in the microscope. The images were further processed by image J to measure the length of the plants and the perimeter of the leaves. Perimeters of the “leaves” grown in BCD were measured by drawing a line from the base of the stem to the tip of the leaf using the freehand tool function of Image J to obtain the “leaf” area. Pictures and all data used for these measurements can be found here at this repository: https://doi.org/10.11583/DTU.20050499.

### Cell biomass measurement

Following this, a second experiment was conducted also with a single gametophore from the *ZmUbi:PpCDKA1–PpCDKA2–OsAct:PpCYCD2–PpCYCD1* line and the WT was grown in 6 well microplate in liquid BCD media. After 90 days of cultivation, cells were dried at 65 °C for 24 h and weighed for dry weight measurements.

### qRT-PCR analysis

RNA transcription levels *for ZmUbi:PpCDKA1, ZmUbi:PpCDKA2, ZmUbi:PpCYCD1, ZmUbi:PpCYCD2, ZmUbi:PpCDKA1–PpCDKA2–OsAct:PpCYCD2–PpCYCD1* and WT lines were measured by extracting the total RNA from cell lines (in triplicates) after 10 days of inoculation in BCD and BCDAT liquid mediums, using Spectrum™ Plant Total RNA Kit (Sigma, STRN250). A total of 500 ng of each RNA sample was reverse transcribed to cDNA with a iScriptTM cDNA Synthesis kit (BIO-RAD, Copenhagen, Denmark). RT-PCR was performed using QuantiFast^®^ SYBR^®^ Green PCR (Qiagen, Copenhagen, Denmark) according to the manufacturer’s protocol. The following amplification program was used: 95 °C 5 min, 40 cycles at 95 °C 10 s followed by 60 °C 30 s. Samples were amplified in duplicate from the same RNA isolation. RT-PCRs were performed using three biological triplicates with qPCR primers designed for the necessary genes and the actin and tubulin controls (Supplementary Table S5). RT-PCR efficiency, E, was estimated for each gene by generating standard curves by plotting quantification cycle (Cq) values (y) against the log of a serial cDNA dilution (x). For this, a cDNA sample was used as a template in a range of 25, 5, 2.5, 1.2 and 0.6 ng. The RT-PCR efficiencies were calculated from the slope of the linear regression equations of the standard curves, along with the regression coefficient (R2). The equation used was E = 10(–1/a), E values in a range of 1.90–2.10 (PCR efficiency between 90 and 110%) with a regression coefficient below 0.02 are acceptable. The ectopic expression of the four genes was also verified (Supplementary Fig. S5).

### Cell wall and starch composition

All the transgenic lines and WT lines were grown in triplicates in BCD medium, changed every two weeks, for 6 weeks and freeze-dried for 48 h. Dried samples were ball milled in 70% EtOH in a Genogrinder (SPEX, NJ, USA) for 2 min. at 1500 rpm, centrifuged for 10 min. at 10,000*g* and the supernatant was discarded. The samples were first extracted with chloroform/MeOH (1:1 v/v) by adding the chloroform/MeOH, vortexing vigorously followed by centrifugation at 10,000*g* for 10 min. The supernatant was discarded and the pellet was extracted with 100% acetone as above. The supernatant was again discarded and the samples were then left to air dry in the fume hood. The sample weight was noted and starch was digested as described in Mosele et al., 2011 with Termamyl (Novozymes, Denmark) and amyloglucosidase (Megazyme, Ireland). The EtOH wash containing the Glc, maltose, and smaller dextrins, was dried in a speed-vac and the pellet was hydrolysed in 2 M TFA as previously described^30^ and the glucose quantified using a Dionex ICS 5000 + DC system (ThermoFisher, MA, USA) as described in Jensen et al., 2018. The pellet after starch removal was dried and analysed by both CoMPP as previously described^31,32^, and by sugar composition analysis after TFA hydrolysis as described above for starch.

### GC/MS analysis of the* ent-kaurene*

Blended *ZmUbi:PpCDKA1, ZmUbi:PpCDKA2,* and WT lines were grown for 18 days in BCDAT media. Cell lines were dried at 98 °C for 24 h, and 10 mg of dry matter was extracted with 1 mL of ethyl acetate using a bead beater for 120 s at maximum power with 0.5 g of glass beads. Lysed cells were further extracted with ethyl acetate in an ultrasonic bath for 1 h. Completely extracted cells were centrifuged for 3 min at 12,000×*g* and 0.75 ml of the top layer containing hydrophobic molecules were isolated. 1 µl of the extracted ethyl acetate layer was injected into a GC/MS equipped with a ZB-5 ms column (30 m × 0.25 mm × 0.25 μm). The samples were injected in a non-split mode using the following program: Initial temperature of the oven was held for 1 min at 50 °C. Then, the temperature was increased to 320 °C at 15 °C/min and held at 325 °C for 5 min. The total run took 24 min. The injector temperature was set at 250 °C. The ion source temperature of the mass spectrometer was 230 °C. The data obtained was analysed for *ent*-kaurene and *ent*-kaurenoic acid using the mass spectra data published previously^33,34^.

## Supplementary Information

Supplementary Information.

## Data Availability

Data not represented online are available from the corresponding author upon reasonable request. The genes *PpCDKA1* (AJ515321), *PpCDKA2* (AB547329), *PpCYCD1* (AJ428953) and *PpCYCD2* (AB547332), are available from the NCBI database (https://www.ncbi.nlm.nih.gov/). The data used for broader image analysis, dry weight data, cell wall measurements, images of different setting of moss growth not used in the paper are available in the DTU repository. Simonsen, Henrik Toft (2023): Data for Overexpression of *Physcomitrium patens* Cell Cycle Regulators Leads to Larger Gametophytes. Technical University of Denmark. Dataset. https://doi.org/10.11583/DTU.20050499.
