# Supplementary material for: Overexpression of Physcomitrium patens cell cycle regulators leads to larger gametophytes
Source: Sci Rep. 2023 Mar 15;13:4301. doi: 10.1038/s41598-023-31417-x (PMC10017697; doi:10.1038/s41598-023-31417-x)
Supplement: Supplementary file 1 — Supplementary Information. [file 41598_2023_31417_MOESM1_ESM.pdf]

Supplementary Figures

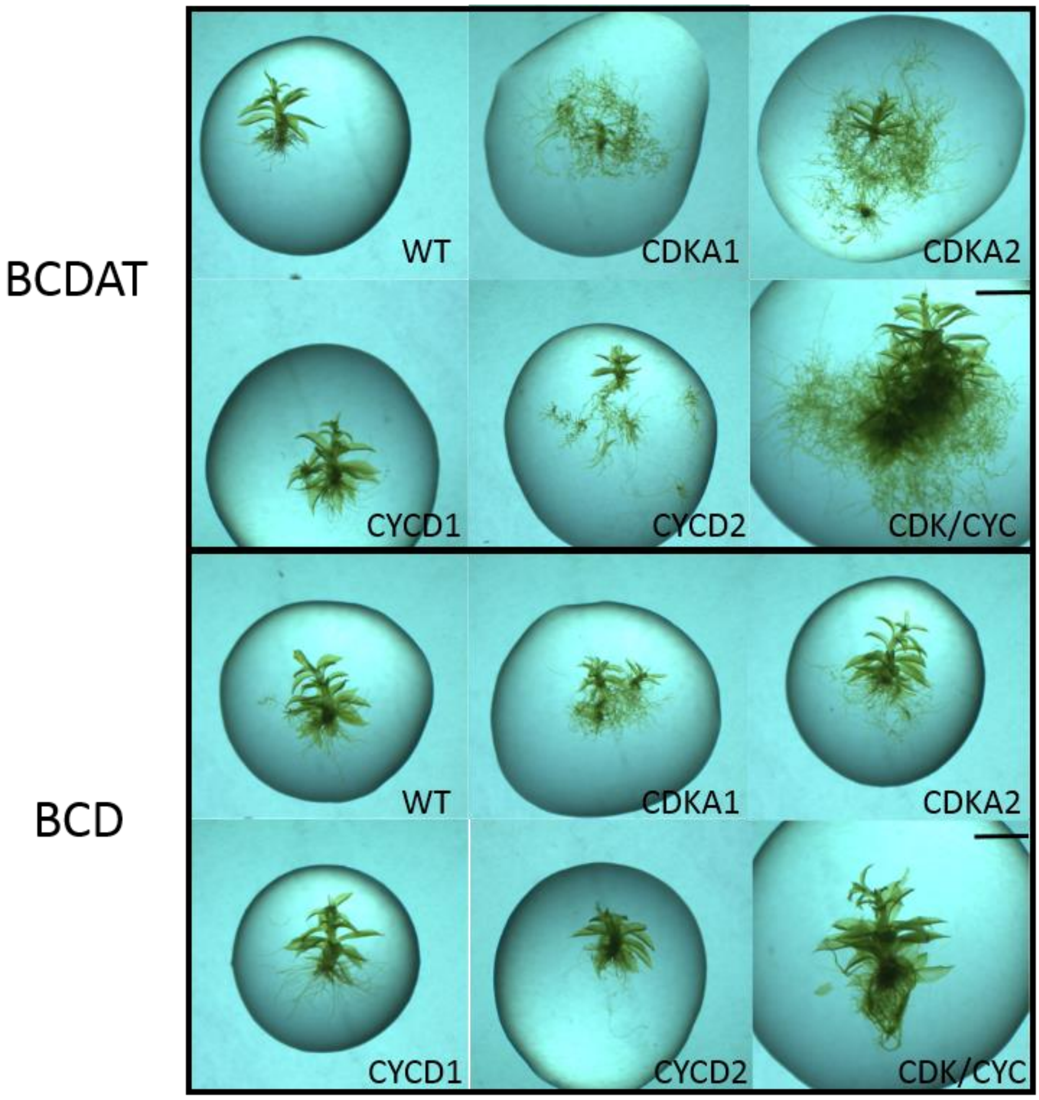

Figure S1. Zoom in to show the physiology of gametophores grown in continuous BCDAT and BCD liquid media for 4 weeks after homogenizing. Scale bar = 0.6 mm

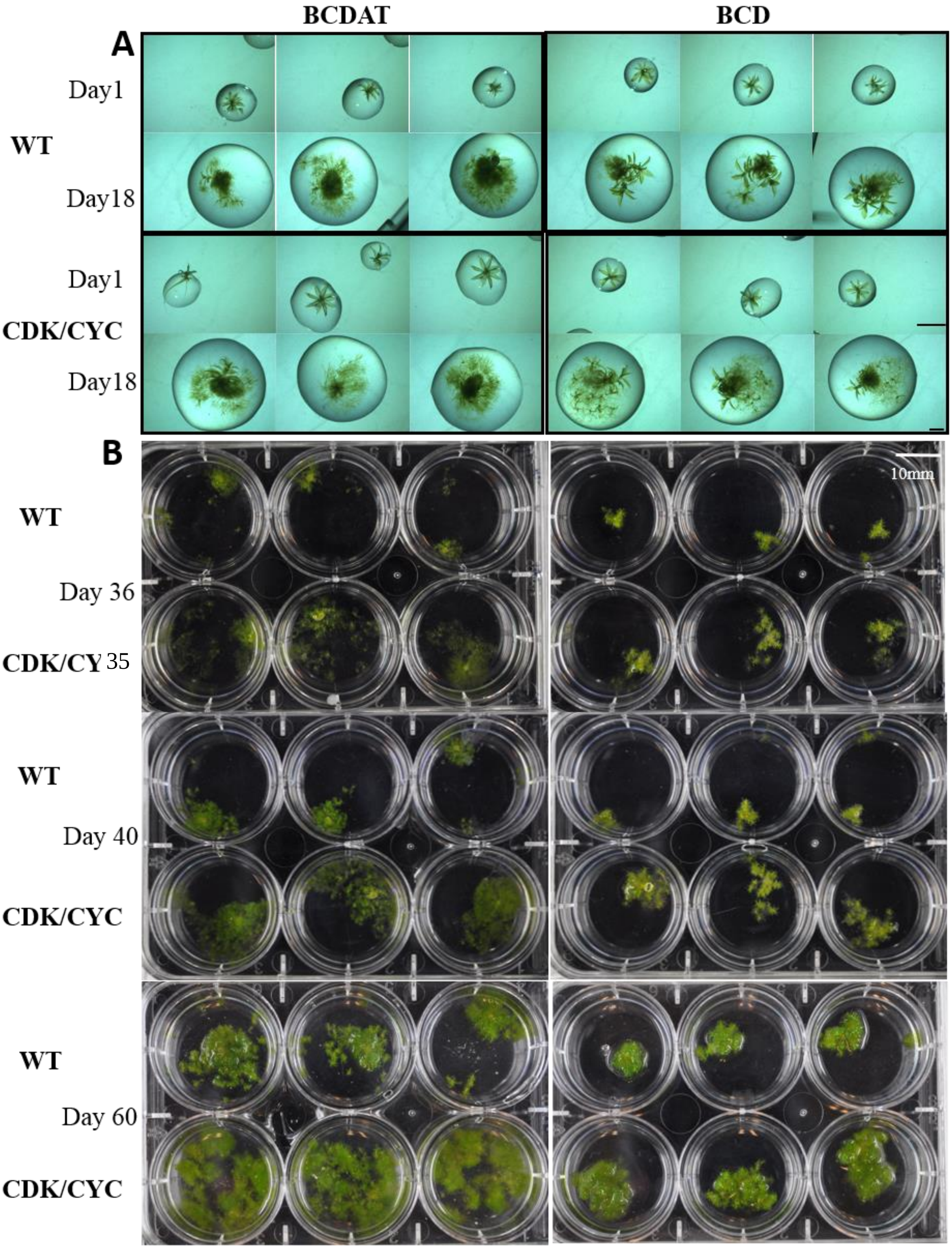

Figure S2. A) Triplicates of gametophores with similar heights and rhizoids from WT and *ZmUbi:PpCKA1-PpCDKA2-OsAct:PpCYCD1-PpCYCD2* (CDK/CYC) lines were isolated from cell lines grown in solid agar BCD medium (n = 3) and inoculated in liquid BCDAT and BCD medium. Images are visualized with a Miotic SMZ-171 microscope and imaged using a Moticam 5.0 MP camera. Scale bar = 1.5 mm. B) Triplicates of cells grown further and photographed using a Nikon 850 camera. Scale bar = 10 mm

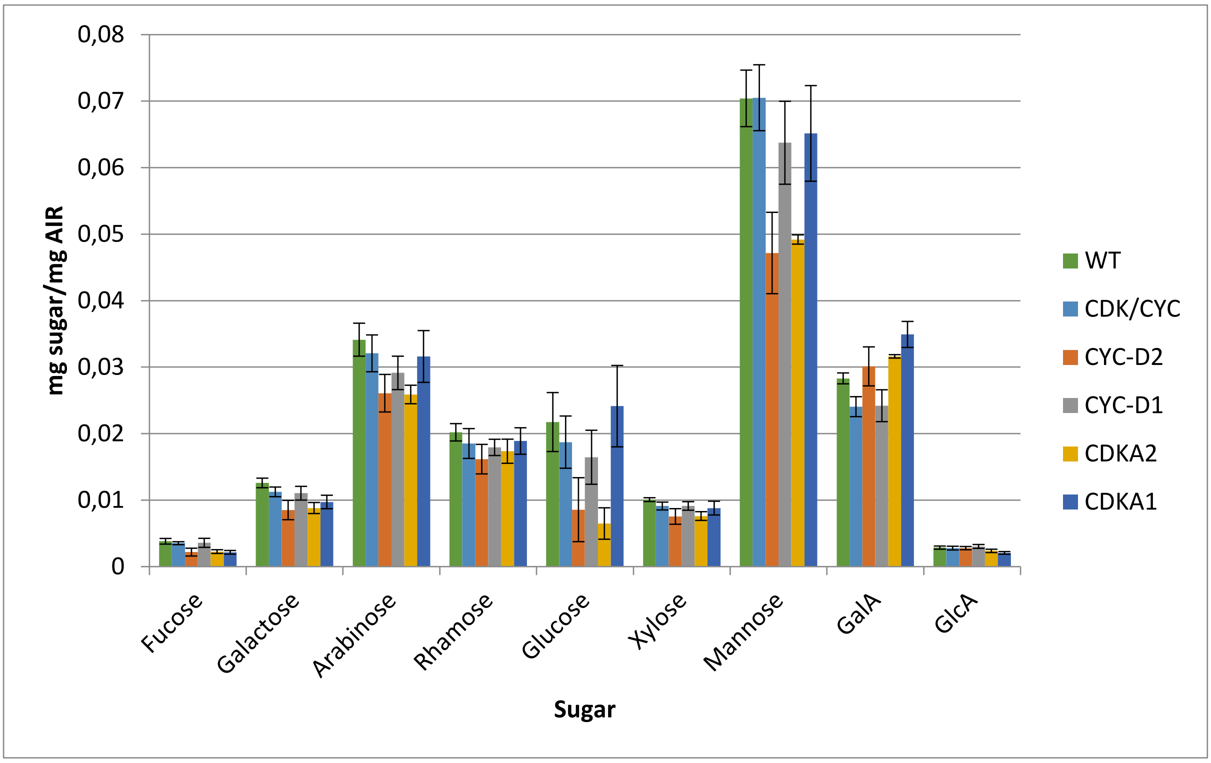

Figure S3: **Cell wall composition analysis** of destarched alcohol insoluble residue (AIR) from WT and engineered Pp. All measurements were done in triplicates. Compared to WT, CYC-D2 and CDKA2 showed significantly lowered content of Glc and Man (Tukey test: Glc; CYC-D2 p=0,031, CDKA2 p=0,012) (Man; CYC-D2 p=0,002, CDKA2 p=0,004) with subsequent lowered total amount of sugar in AIR. This reduction is putatively due to reduction of glucomannan content of the cell wall, as (gluco)mannan is a prominent component of Pp cell walls (Moller et al., 2007). The CoMPP analysis do not show any difference in (galacto)(gluco)mannan (LM21), but the (galacto)(gluco)mannan could be of a structure not recognised by LM21 or not extractable with 4M NaOH as done in CoMPP.

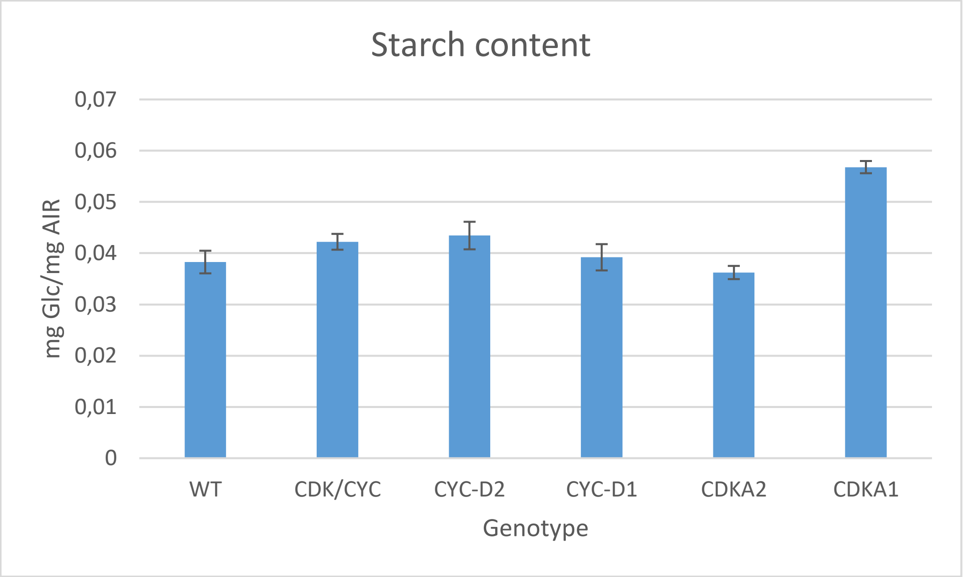

Tukey test				
Category	Groups			p difference to WT
CDKA1	A			< 0,0001
CYC-D2		B		
CDK/CYC		B		
CYC-D1		B	C	
WT		B	C	
CDKA2			C	

Figure S4: Starch content of mutant lines. None of the engineered Pp strains shows a reduced starch content, when compared to WT. CDKA1 actually contain higher amounts of starch, compared to WT and the other strains.

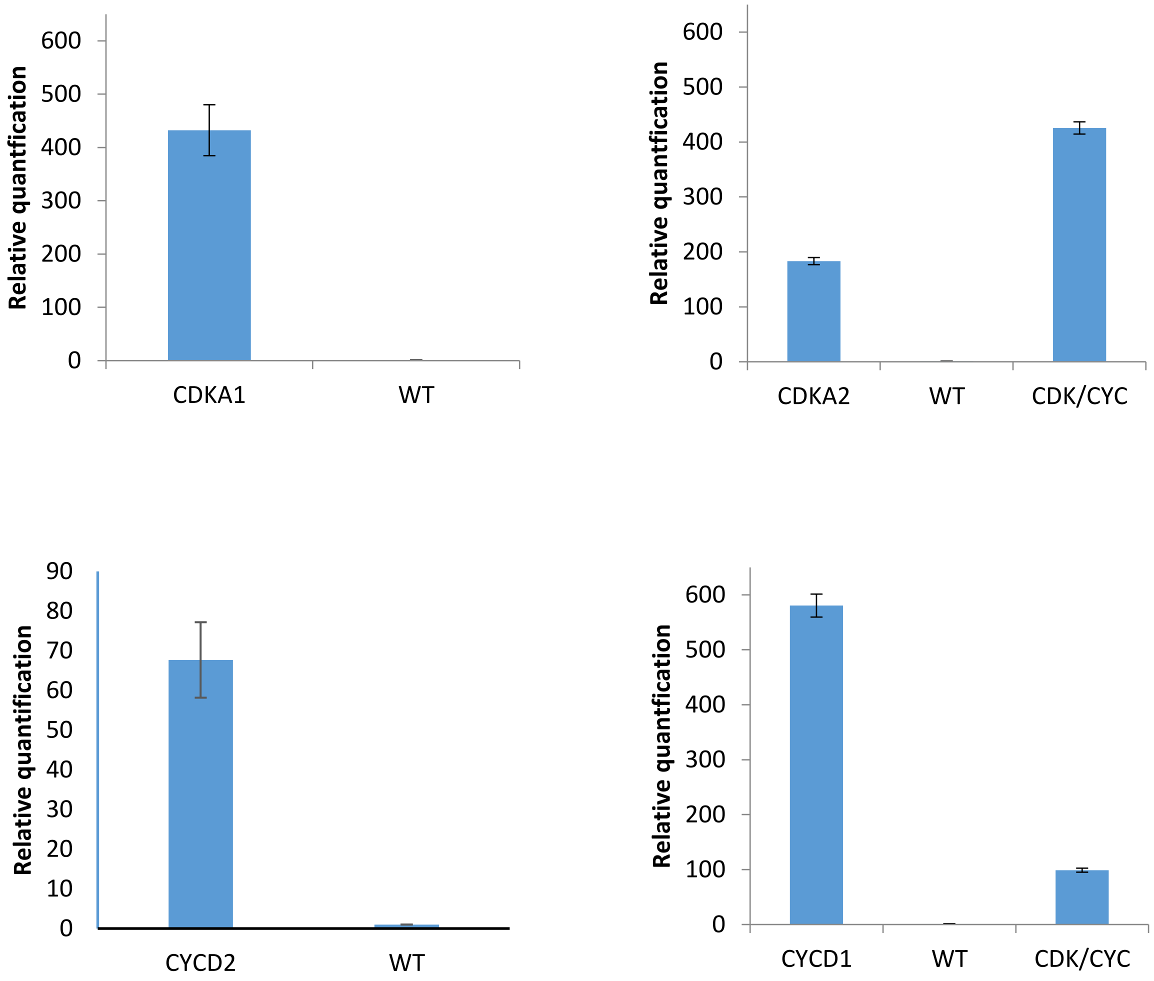

Figure S5. Verification of ectopic expression of CDKA1, CDKA2, CYCD1 and CYCD2 through qRT-PCR. All overexpressed genes showed a high expression level. Both CYCD2 and CYCD1 and was transfected as one fragment in the *ZmUbi:PpCKA1-PpCDKA2-OsAct:PpCYCD2-PpCYCD1* line. Thus, the verification of the expression was confirmed on the last gene of the fragment (CYCD1). Similar verification process was followed on CDKA1-CDKA2. All measurements were done in triplicates.

Supplemental Tables

Extraction	Sample	Sample	HG partially/de-esterified (mAb LM18)	HG partially/de-esterified (mAb LM19)	(1→4)-β-D-galactan (mAb LM5)	(1→5)-α-L-arabinan (mAb LM6)	Linearised (1→5)-α-L-arabinan (mAb LM13)	(1→4)-β-D-(galacto)mannan (mAb BS-400-4)	(1→4)-β-D-(galacto)(gluco)mannan (mAb LM21)	(1→3)(1→4)-β-D-glucan (mAb BS-400-3)	Processed (1→5)-α-L-arabinan (mAb LM16)	Xyloglucan (XXXG motif) (mAb LM15)	Xyloglucan / unsubstituted β-D-glucan (mAb LM25)	(1→4)-β-D-xylan (mAb LM10)	(1→4)-β-D-xylan/arabinoxylan (mAb LM11)	Anti-grass xylan preparations (mAb LM27)	Glucuronoxylan (mAb LM28)	Extensin (mAb JIM20)	AGP (mAb JIM13)
CDTA	1	CDK/CYC	6	0	0	25	0	97	54	0	0	0	0	0	0	0	0	0	0
CDTA	2	CYC-D2	9	6	0	29	0	90	51	7	0	0	0	0	0	0	0	0	0
CDTA	3	CYC-D1	7	0	0	26	0	100	56	0	0	0	0	0	0	0	0	0	0
CDTA	4	CDKA2	11	7	6	28	0	84	45	0	0	0	0	0	0	0	0	0	0
CDTA	5	CDKA1	23	16	0	24	0	98	60	0	0	0	0	0	0	0	0	0	0
CDTA	6	WT	9	6	0	29	0	89	51	0	0	0	0	0	0	0	0	0	0
NaOH	1	CDK/CYC	0	0	0	15	0	57	31	0	0	0	19	0	0	0	0	0	0
NaOH	2	CYC-D2	0	0	0	17	0	50	27	0	0	0	21	0	0	0	0	0	0
NaOH	3	CYC-D1	0	0	0	16	0	55	30	0	0	0	20	0	0	0	0	0	0
NaOH	4	CDKA2	0	0	0	14	0	49	26	0	0	0	19	0	0	0	0	0	0
NaOH	5	CDKA1	0	0	0	11	0	46	24	0	0	0	16	0	0	0	0	0	0
NaOH	6	WT	0	0	0	15	0	55	29	0	0	0	19	0	0	0	0	0	0

Table S1: CoMPP analysis. Beside CDKA1 showing a slightly different labelling of homogalacturonan (HG), indicating a slightly lowered degree of methylation of the homogalacturonan, no differences between WT and engineered strains could be observed.

Primer		Sequence 5'-3'
pRH 4.7 kb	F	CCAGATCGACCACATCCTTCTCCG
pRH 4.7 kb	R	GACCTGCAGAAGTAACACCAAACAACAG
pRH 2.1 kb	F	GTCCTGCTTTAATGAGATATGCGAGACG
pRH 2.1 kb	R	ACGAAGGCCGTTCTTCCCTG
CDKA1	F	CTGTTGTTTGGTGTTACTTCTGCAGGTCATGGATCAGTATGAGAAAGTGG
CDKA1	R	CGTCTCGCATATCTCATTAAAGCAGGACTCAGGGTACAAGACCGATATC
CDKA2	F	CTGTTGTTTGGTGTTACTTCTGCAGGTCATGGAACAGTATGAAAAAGTGG
CDKA2	R	CGTCTCGCATATCTCATTAAAGCAGGACTCACGGCACCAGACC
CYCD1	F	CTGTTGTTTGGTGTTACTTCTGCAGGTCATGTCTCCCAGTGTTGATTG
CYCD1	R	CGTCTCGCATATCTCATTAAAGCAGGACTTATAGGCTGGCGCTCTCTA
CYCD2	F	CTGTTGTTTGGTGTTACTTCTGCAGGTCATGTCGCCCAGTGTTGA
CYCD2	R	CGTCTCGCATATCTCATTAAAGCAGGACTCATAGGTGGGTTCTCTCTAACA

Table S2: Primers for individual gene overexpression. pRH 4.7 kb PCR fragment is consisted of 108-5' neutral genome integration region, G418 antibiotic selection marker driven by CaMV 35S promoter and the *Zea mays* ubiquitin1 promoter. pRH2.1 kb fragment is consisted of the OCS terminator and 108-3' neutral genome integration region. All the transformation for individual gene overexpression was done by transfecting *P. patens* cells with three PCR fragments of pRH 4.7 kb, pRH 2.1 kb and the CDS of the appropriate gene.

Primer		Sequence 5'-3'
pRH 4.7 kb	F	CCAGATCGACCACATCCTTCTCCG
pRH 4.7 kb	R	GACCTGCAGAAGTAACACCAAACAACAG
CDKA1/CDKA2	F	GTTACTTCTGCAGGTATGGATCAGTATGAGAAAGTGGAGAAGATTG
CDKA1/CDKA2	R	ATCTCATTAAAGCAGTCACGGCACCAGACCGAC
OCS terminator	F	GGTCTGGTGCCGTGACTGCTTTAATGAGATATGCGAGAC
OCS terminator	R	GAATGACCTCGGGCCCTGCTGAGCCTCGACATG
Rice Actin	F	GTCGAGGCTCAGCAGGGCCCGAGGTCATTCATATG
Rice Actin	R	GATCCCCGATATCTTCTAC
CYCD2/CYCD1	F	GGATCCCCGATATCTTCTACCTAC
CYCD2/CYCD1	R	GTTTGAACGATCGGCCG
NOS Terminator	F	CGGCCGATCGTTCAAACATTTGG
NOS Terminator	R	CTCAAAAAGAAAGAATTAGATCTAGTAACATAGATGACAC
108_3'_locus	F	CTATGTTACTAGATCTAATTCTTTCTTTTTGAGGTATATATTATCTTA GCATGG
108_3'_locus	R	ACGAAGGCCGTTCTTCCC

Table S3: Primers for all four genes overexpression. Seven fragments were PCR amplified and transfected to *P. patens* cells to overexpress all four CDK/CYC in the same line. The synthesized *PpCDKA1-PpCDKA2* construct was driven by *ZmUbi1* promoter and OCS terminator and the synthesized *PpCYCD2-PpCYCD1* fragment was driven by *OsActin* promoter and NOS terminator.

Genotype Primers		Sequence 5'-3'
ZmUbi	F	TGTCCTTAATCTTGTGCTAGTTCT
OCS R	R	CAACGTGCACAACAGAATTGAAAGC
Rice Actin	F	ATTTGTGACAAATGCAGCC
NOS	R	CTTTATTGCCAAATGTTTGAAC
108-5' Integration	F	CATCAACAAATCAAGGAGTCA
108-5' Integration	R	CCACCATGTTTGAGTTGG
108-3' Integration	F	GAAACAGGCCTAGCAGAAA
108-3' Integration	R	CACATGTGGAATTCTACTAATACAA

Table S4: Genotype Primers. Stable integration of the fragments in proper orientation to Pp108 neutral locus was confirmed using the genotype primers.

qRT-PCR Primer		Sequence 5'-3'
CDKA1	F	TTGCCAGATTTCAAGACTGC
CDKA1	R	GTGTTCCAATGCATTTCGTG
CDKA2	F	GTGGCCTGCAAAGAATGTGG
CDKA2	R	TCGTGCTCCAATGCAGTTCT
CYCB1	F	CGTGCTGTCTCCGGTCTCTT
CYCB1	R	CCTGGTAGCGAACATGGTTGT
CYCB2	F	TCCGAGGCTCAGATCAAGGA
CYCB2	R	TCACTGGCCTTGCTGTGAAG
CYCD1	F	AGTATTGGAGGCAGCCGATG
CYCD1	R	GAGACAACTTGCGACGCTTG
CYCD2	F	CCGCAGAGCCCTATTGGA
CYCD2	R	CTTCAGTGGCGGAGCTCAA
KRP	F	TCCCCGCGATCTCCATTAC
KRP	R	GTTGAAGTAGCGCACGACACTT
HFO	F	GCTCGTCGTGGTGGAGTGA
HFO	R	CGCCTCGAGTCTCCTCGTAT
RSL2	F	GGGACCTCAAGGATGCAGCA
RSL2	R	CGAACTCAATAACGTCAGGA
PINA	F	TCCAGGAAGCCAAACAGCCAT
PINA	R	CTCTGCCAGTTTCGGTGTCAA
PINB	F	GTCTTGTTACTCCCGGAGGTA
PINB	R	CTTTGCTTCGTCTTCGGGTA
α-tubulin	F	CGTAGGAGGGACCAGTTTGG
α-tubulin	R	TGCATTCATCCCCGAGTCA
Actin	F	GGACTTCGAGCAGGAGTT
Actin	R	TCATTATCGAGTTGTACGTCG

Table S5: qRT-PCR primers. The qRT-PCR primer sequences used in this study. All the expression data was done in biological triplicates and technical duplicates with the subsequent normalization of the transcript levels to actin and α-tubulin
